# Distinct maturation, glucose metabolism, and inflammatory function of human monocytes-derived IDECs mediated by anti-IgE and Pam3CSK4 alone or in combination

**DOI:** 10.3389/fimmu.2024.1403263

**Published:** 2024-07-17

**Authors:** Cuie Gao, Ying Zhao, Lan Ge, Wenying Liu, Mengjie Zhang, Bing Ni, Zhiqiang Song

**Affiliations:** ^1^ Department of Dermatology, Southwest Hospital, Army Medical University, Chongqing, China; ^2^ Department of Pathophysiology, College of High Altitude Military Medicine, Army Medical University, Chongqing, China

**Keywords:** atopic dermatitis, high-affinity IgE receptor, TLR2, metabolism, glycolysis

## Abstract

**Background:**

Cell energy metabolism controls the activation and function of dendritic cells (DCs). Inflammatory dendritic epidermal cells (IDECs) in skin lesions of atopic dermatitis (AD) express high-affinity IgE receptor (FcϵRI) and toll-like receptor 2 (TLR2), which mediate the generation and maintenance of inflammation. However, cellular energy metabolism and effector function of IDECs mediated by FcϵRI and TLR2 have not been fully elucidated.

**Methods:**

IDECs *in vitro* were treated with TLR2 agonist Pam3CSK4 and anti-IgE alone or in combination for 24 h. Further, we analyzed the expression of cell surface activation markers, production of inflammatory factors, and cellular energy metabolism profiles of IDECs by using flow cytometry, multiplex assay, RNA sequencing, targeted energy metabolism, and seahorse assays.

**Results:**

Compared to the unstimulated or anti-IgE groups, Pam3CSK4 alone or combined with anti-IgE groups significantly increased the expression of CD80, CD83, and CD86 on IDECs, but did not affect the expression of the above markers in the anti-IgE group. The release of inflammatory cytokines increased in the Pam3CSK4 alone or combined with anti-IgE groups, while there was a weak increasing trend in the anti-IgE group. The glycolysis/gluconeogenesis pathway of carbon metabolism was affected in all treatment groups. Furthermore, compared to the control group, we found a decrease in pyruvic acid, upregulation of *PFKM*, downregulation of *FBP1*, and increase in extracellular lactate, glycolysis rate, and glycolysis capacity after all treatments, while there was no difference between each treatment group. However, there was no difference in glycolytic reserve and mitochondrial basic and maximum respiration among all groups.

**Conclusion:**

Our results indicate that glycolysis of IDECs may be activated through FcϵRI and TLR2 to upregulate inflammatory factors, suggesting that danger signals from bacteria or allergens might evoke an inflammatory response from AD through the glycolysis pathway.

## 1 Introduction

Atopic dermatitis (AD) is a common chronic inflammatory skin disease characterized by recurrent itching and dryness of the skin. AD affects up to 20% of children and 10% of adults in high-income countries (1). The pathophysiology of AD is complex due to the interaction of multiple factors, including genetic and environmental factors, microbial imbalance, immune abnormalities, and skin barrier dysfunction, which drive skin inflammation (2).

Dendritic cells (DCs) are the major antigen-presenting cells and have been recognized as key bridge cells between the innate and adaptive immune systems (3). The prominent feature of AD is the skin infiltration of DCs including inflammatory dendritic epidermal cells (IDECs) (3, 4), which has received less attention. It has been reported that IDECs are mainly derived from monocytes and express the high-affinity IgE receptor (FcϵRI), and that FcϵRI^+^ IDECs are characteristic inflammatory DCs in AD lesions, which may play an important role in the generation and maintenance of inflammation by inducing different T-cell responses (3–8). In addition, immune cells including DCs undergo different metabolic reprogramming after activation, which not only meets the energy needs of these cells, but also promotes the secretion of inflammatory cytokine and induces the expression of co-stimulatory molecules that initiate acquired immune responses (9–13). Previous studies have shown that the cellular energy metabolism and function of human monocytes-derived DCs are altered after treatment with various factors, such as mechanical stiffness, polymerized allergoids conjugated to mannan and lipopolysaccharide (14–18). Studies have also reported that cellular energy metabolism, such as glycolysis, plays a role in skin inflammation in mice with AD (19–21). For example, increased glycolysis in the epidermis of mice with AD is essential for keratinocyte proliferation (21), and the use of glycolytic inhibitor 2-deoxy-D-glucose (2-DG) could significantly improve skin inflammation in mice with dermatitis (19). However, the cellular energy metabolism and effector function of IDECs mediated through FcϵRI remain elusive in AD.

In addition, toll-like receptor 2 (TLR2) is also expressed on IDECs and mediates the pathogenesis of AD (22–24). IDECs with high expression of both FcϵRI and TLR2 may be stimulated by different exogenous antibodies and/or ligands. Previously, we found that the TLR2 agonist Pam3CSK4 induced the expression of FcϵRI on circulating monocytes in patients with moderate or severe AD through the p38 MAPK signaling pathway (22), suggesting that the interaction between FcϵRI and TLR2 also occurs in AD that needs further verification. Moreover, studies reported that the interaction enhanced the release of inflammatory factors (25–27), so we speculate that the interaction may mediate the cellular energy metabolism and function of IDECs in AD.

Therefore, the current study aimed to analyze the expression of cell surface activation markers, the production of inflammatory factors, and changes in cellular energy metabolism in an *in vitro* model of IDECs treated with Pam3CSK4 and anti-IgE alone or in combination after 24 h.

## 2 Methods

### 2.1 Subjects

Human peripheral blood was collected from healthy volunteers (n = 15) without any skin problems; the individuals recruited were from the Southwest Hospital of the Army Medical University, Chongqing, China in 2022–2023. The study was approved by the Ethics Committee of Southwest Hospital (Approval number KY202258). All study participants agreed to participate in the study and signed an informed consent.

### 2.2 Monocytes isolation and induction of *in vitro* model of IDECs

**Figure 1:**
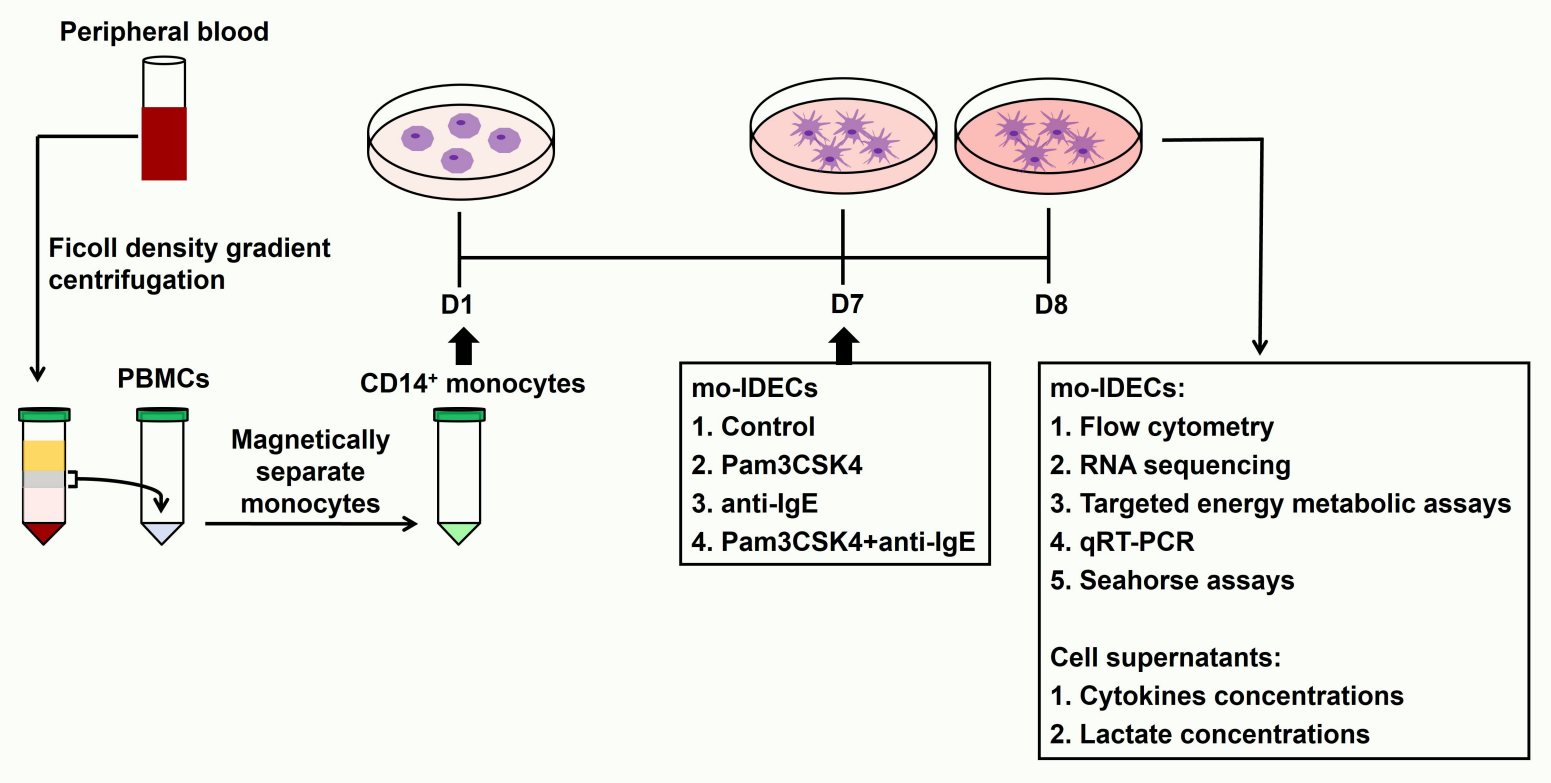
Schematic diagram of the framework of the study design.

Human peripheral blood mononuclear cells (PBMCs) were isolated using density gradient centrifugation by laying blood samples on the Lymphoprep™ (StemCell Technologies, Germany). CD14^+^ monocytes were isolated using magnetic beads (Miltenyi Biotec, Bergisch Gladbach, Germany) according to the manufacturer’s instructions and confirmed by flow cytometry (the purity of CD14^+^ cells was greater than 90%). The *in vitro* cell model of IDECs has been induced as described in previous studies (8, 28) and the induction steps of this model are as follows. We seeded 10^6^ CD14^+^ monocytes/mL IMDM complete medium in 24-well plates. Cells were cultured with human recombinant IL-4 (75 ng/mL, Miltenyi Biotec), GM-CSF (50 ng/mL, R&D Systems, Minneapolis, USA), IgE (1 ug/mL, Abcam, Cambridge, UK) and β-mercaptoethanol (β-ME) (5 mM, Sigma Aldrich, St. Louis, MO). A complete medium (50 uL) containing the above factors was added to each well on day 3 and 500 uL of the supernatant was carefully discarded from each well followed by 500 uL of the complete medium containing the above factors on day 5. On day 7, we exposed mo-IDECs to Pam3CSK4 (1 ug/mL, Tocris Bioscience, Tocris Cookson Ltd., UK), anti-IgE (5 ug/mL, Bethyl Laboratories, USA) or Pam3CSK4 and anti-IgE for 24 h. mo-IDECs (immature mo-IDECs, Pam3CSK4 mo-IDECs, anti-IgE mo-IDECs, and Pam3CSK4 combined with anti-IgE mo-IDECs) and supernatants were collected for subsequent experiments. The whole experiment is shown in a schematic diagram (**Figure 1**).

### 2.3 Flow cytometry

**Figure 2:**
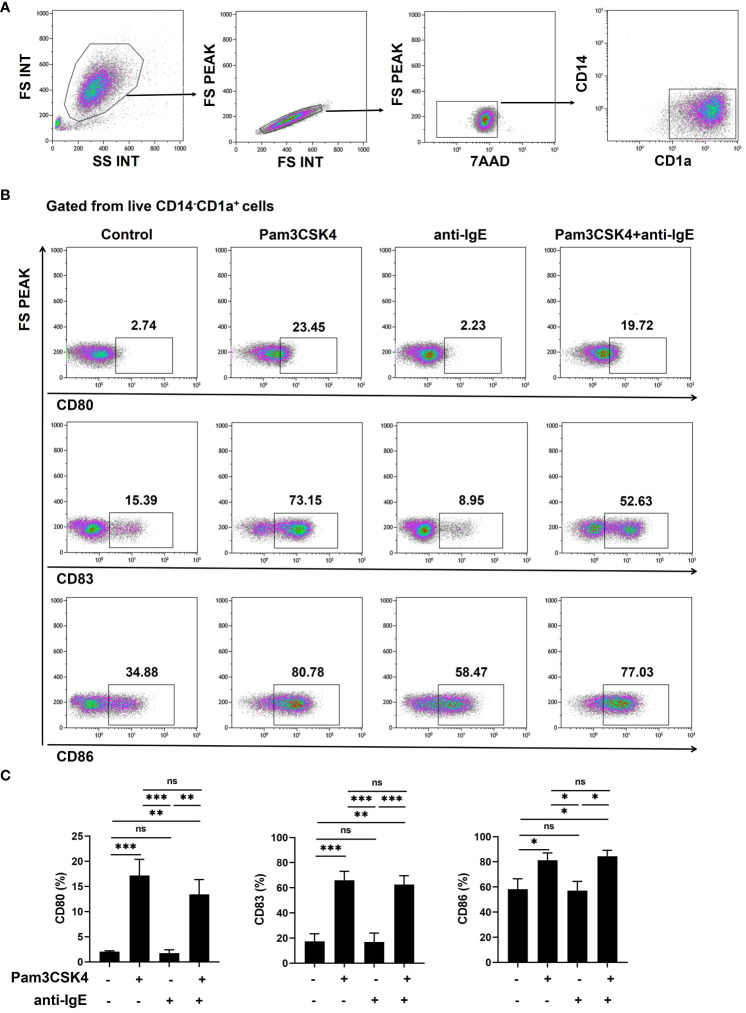
Pam3CSK4 alone or combined with anti-IgE stimulates the maturation of mo-IDECs *in vitro* by flow cytometry. **(A, B)** Specific gating strategies to identify mo-IDECs and CD80, CD83, and CD86 expression on the surface of mo-IDECs after stimulation with different stimulus for 24 h. **(C)** Surface expression of co-stimulatory molecules (CD80, CD83, and CD86) on the surface of live mo-IDEC (CD14^-^CD1a^+^) cells (n = 5–6). Data were presented as mean ± SEM. Statistical significance between the stimulation group and the control group was analyzed by one-way ANOVA. ^ns^ *p* > 0.05, * *p* < 0.05, ** *p* < 0.01, and *** *p* < 0.001. ns represents not significant.

Isolated human CD14^+^ monocytes were stained with CD14 antibody (Brilliant Violet 510™, BioLegend, San Diego, CA, USA) and mo-IDECs with CD14 (Brilliant Violet 510™), CD1a (PE/Cyanine7), CD80 (FITC), CD83 (PE), CD86 (Brilliant Violet 421™), and FcϵRIα (Brilliant Violet 510™) antibodies. Dead mo-IDECs were excluded with 7-AAD viability staining solution. All antibodies were obtained from BioLegend (San Diego, CA, USA). Flow cytometry was performed with Navios Flow Cytometer (Beckman Coulter, Miami, USA) and data were analyzed using the Navios Software version 1.3. Live CD14^-^CD1a^+^ cells were identified as mo-IDECs. Specific gating strategies to identify CD80, CD83, CD86, and FcϵRIα expression in mo-IDECs are shown in **Figures 2A, B** and **Supplementary Figure S1A**.

### 2.4 Measurement of cytokines

Secretion of cytokines by mo-IDECs was analyzed in cell-free supernatants using a Human Inflammation Panel 1 with V-bottom Plate from BioLegend according to the manufacturer’s instructions. Data were acquired from Navios Flow Cytometer (Beckman Coulter) and analyzed using the software provided by BioLegend.

### 2.5 RNA sequencing

Total RNA was extracted from mo-IDECs using RNAiso Plus (TaKaRa, Dalian, China). Sequencing libraries were constructed using NEBNext^®^ Ultra™ RNA Library Prep Kit for Illumina^®^ and subsequently sequenced using the Illumina NovaSeq PE150 sequencing strategy. Four biological repeats were collected in each group. The reference *Homo Sapiens* genome and gene information were downloaded from the GRCh38.98 version of the Ensemble database. Raw data were filtered to produce clean data for subsequent data analysis. The expression of RNA-seq gene was measured by Fragments Per Kilobase of exon per Million fragments mapped (FPKM). Differentially expressed genes (DEGs) were identified with *p*-value < 0.05 and absolute value of log_2_ FC (fold change) > 0. Among them, upregulated DEGs were identified with log_2_ FC > 0, while downregulated DEGs were identified with log_2_ FC < 0. The volcano plots and heat maps were analyzed and visualized using R language. Kyoto Encyclopedia of Genes and Genomes (KEGG) was used for pathway enrichment of upregulated or downregulated DEGs, respectively. Finally, the significance of KEGG was determined by a hypergeometric test (*p* values).

### 2.6 Metabolic assays

mo-IDECs were collected and analyzed for targeted energy metabolites based on the ABSciex QTRAP^®^ 6500+ LC-MS/MS platform at MetWare Biotechnology Co., Ltd (Wuhan, China). Five biological replicates were conducted per group. The sample was thawed on ice and 100 μL of ultrapure water extract was added to resuspend the cell pellet. Ice-cold methanol (200 μL) was divided into 50-μL cell suspensions and vortexed for 2 min at 2,500 rpm/min. The sample was frozen in liquid nitrogen for 5 min, removed from ice for 5 min, and vortexed for 2 min. The previous step was repeated three times. The sample was centrifuged at 12,000 rpm/min for 10 min at 4°C. Then, 200 μL of the supernatant was taken into a new centrifuge tube and placed in -20°C refrigerator for 30 min. The supernatant was centrifuged at 12,000 rpm/min for 10 min at 4°C. After centrifugation, 180 μL of the sample extracts was analyzed based on the LC-ESI-MS/MS system (Waters ACQUITY H-Class, https://www.waters.com/nextgen/us/en.html; MS, QTRAP^®^ 6500+ System, https://sciex.com/). The system is equipped with an ESI turbo ion–spray interface, operated in both positive and negative ion modes and controlled by the Analyst 1.6 software (AB Sciex). The left extracts were frozen and thawed three times, centrifuged at 12,000 rpm/min for 10 min, and then, the supernatant was taken to determine the protein concentration by BCA Protein Assay Kit. Differential metabolites between groups were identified with VIP ≥ 1 and fold change ≥ 2 or fold change ≤ 0.5. The differential metabolites among the different treatment groups were represented by heat maps, which were analyzed and visualized using R language. Identified metabolites were annotated using the KEGG compound database (http://www.kegg.jp/kegg/compound/) and the annotated metabolites were then mapped to the KEGG Pathway database (http://www.kegg.jp/kegg/pathway.html) and their significance was determined by a hypergeometric test (*p* values).

### 2.7 Analysis of extracellular lactate concentrations

Lactate measurements of mo-IDECs culture supernatants were determined by using the Lactate Assay Kit (Sigma Aldrich).

### 2.8 RNA extraction and quantitative real-time PCR

Total RNA was extracted according to the instructions of RNAiso Plus (TaKaRa). For *PFKL*, *PKFM*, *PFKP*, and *FBP1* detection, cDNAs were synthesized using PrimeScript™ RT Reagent Kit (TaKaRa). qRT-PCR was performed with TB Gerrn™ Premix Ex Taq™ II (TaKaRa) on a CFX Connect™ Real-Time System (BIO-RAD, Singapore, USA). The expression levels were calibrated to the *β-actin* and determined by the 2^-ΔΔCt^ method. All primers used are shown in **Table 1**.

**Table 1:** qRT-PCR primers used in the current study.

Gene name	Primers’ sequence (5’ - 3’)
*PFKL*	Forward: CAGTTGGCCTGCTTGATGTTCTCA Reverse: GGCATTTATGTGGGTGCCAAAGTC
*PFKM*	Forward: GAGTGACTTGTTGAGTGACCTCCAGAAA Reverse: CACAATGTTCAGGTAGCTGGACTTCG
*PFKP*	Forward: CGGAAGTTCCTGGAGCACCTCTC Reverse: AAGTACACCTTGGCCCCCACGTA
*FBP1*	Forward: TCAACTGCTTCATGCTGGAC Reverse: CGTAGACCAGAGTGCGATGA
*β-actin*	Forward: CTCTTCCAGCCTTCCTTCCT Reverse: AGCACTGTGTTGGCGTACAG

### 2.9 Seahorse assays

mo-IDECs were attached to culture plates at a density of 1.2 × 10^5^ cells per well. Extracellular acidification rate (ECAR) and oxygen consumption rate (OCR) were measured on an Agilent Seahorse XF96 metabolic flux analyzer using a Glycolysis Stress Test Kit (Agilent Technologies, USA) and Cell Mitochondrial Stress Test Kit (Agilent Technologies), respectively. The ECAR was programmed to take three repeated measurements at baseline (measurements 1–3) and following the injection of 10-mM glucose (measurements 4–6), 1-μM oligomycin (measurements 7–9), and 50-mM 2-deoxyglucose (measurements 10–12). The rate of glycolysis and glycolytic capacity were calculated by subtracting glucose-induced ECAR (6th measurement) and oligomycin-induced ECAR (8th measurement) from the baseline (3rd measurement). Glycolytic reserve was calculated by subtracting glycolytic capacity from glycolysis. The OCR was programmed to take three repeated measurements at baseline (measurements 1–3) and following the injection of 2.5-μM oligomycin (measurements 4–6), 2-μM carbonyl cyanide-4 (trifluoromethoxy) phenylhydrazone (FCCP) (measurements 7–9), and 0.5-μM rotenone/antimycin A (R/A) (measurements 10–12). Mitochondrial basal respiration and maximal respiratory capacity were calculated by subtracting the basal OCR (3rd measurement) and maximal OCR (8th measurement) from the baseline (12th measurement).

### 2.10 Statistical analysis

Data were recorded and analyzed with SPSS version 17.0, GraphPad Prism version 5, and R version 3.5.1. Results were expressed as mean ± standard error of the mean (SEM). One-way ANOVA was used to compare the two groups. Differences between the two groups were considered statistically significant at * *p* < 0.05, ** *p* < 0.01, *** *p* < 0.001, and **** *p* < 0.0001.

## 3 Results

### 3.1 Promoting the inflammatory function of mo-IDECs treated with Pam3CSK4 and anti-IgE alone or in combination

To investigate whether mo-IDECs can respond to stimulation with Pam3CSK4 and anti-IgE alone or in combination, we analyzed the expression of maturation markers (CD80, CD83, and CD86) on the surface of mo-IDECs by flow cytometry. Compared to the unstimulated group, Pam3CSK4 alone or in combination groups significantly increased the expression of CD80, CD83, and CD86 to varying degrees, while no major differences were observed in the anti-IgE group (**Figure 2C**). Compared to the Pam3CSK4 alone group, the expression of maturation markers in Pam3CSK4 combined with the anti-IgE group did not change, but significantly increased compared to the anti-IgE alone group (**Figure 2C**). These results showed that Pam3CSK4 mediated the maturation of mo-IDECs, while anti-IgE did not.

**Figure 3:**
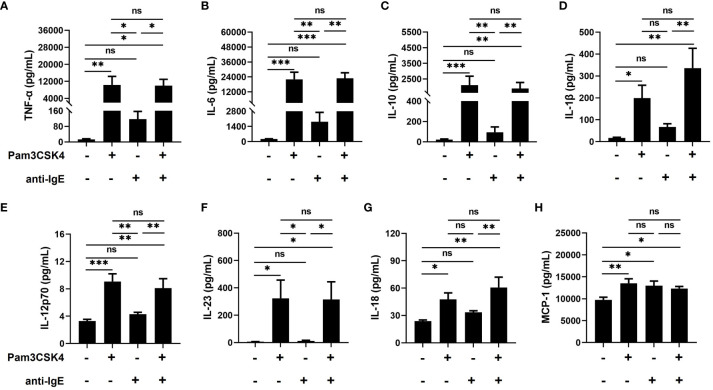
Promoting the inflammatory function of mo-IDECs treated with Pam3CSK4 alone or combined with anti-IgE *in vitro* by multiplex assay. **(A–H)** Levels of cytokines (TNF-α, IL-6, IL-10, IL-1β, IL-12p70, IL-23, IL-18, and MCP-1) in culture supernatants of mo-IDECs after stimulation with different stimulus for 24 h (n = 5–6). Data were presented as mean ± SEM. Statistical significance between the stimulation group and the control group was analyzed by one-way ANOVA. ^ns^ *p* > 0.05, * *p* < 0.05, ** *p* < 0.01, and *** *p* < 0.001. ns represents not significant.

Next, to further assess the effect of Pam3CSK4 and anti-IgE alone or in combination on the inflammatory function of mo-IDECs, we analyzed the release of cytokines, including TNF-α, IL-6, IL-10, IL-1β, IL-12p70, IL-23, IL-18, and monocyte chemotactic protein-1 (MCP-1). Compared to the unstimulated group, mo-IDECs displayed markedly enhanced production of all tested inflammatory cytokines in Pam3CSK4 alone or in combination groups, and the anti-IgE group only significantly stimulated the release of MCP-1, while other cytokines showed a weak increasing trend (**Figure 3**). Compared to the Pam3CSK4 group, the release of inflammatory cytokines in the Pam3CSK4 combined with the anti-IgE group showed no difference, but significantly increased the generation of other inflammatory cytokines except MCP-1 compared to the anti-IgE group (**Figure 3**). These data suggested that Pam3CSK4 modulated the inflammatory effector function (in terms of cytokine secretion) of mo-IDECs more than anti-IgE.

### 3.2 Transcriptome profiling of mo-IDECs

**Figure 4:**
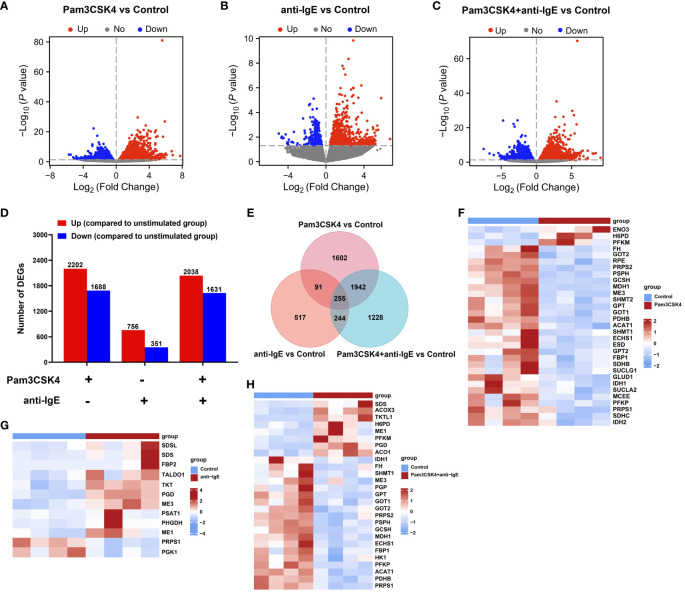
Overview of the transcriptome profiling of mo-IDECs in response to Pam3CSK4 and anti-IgE alone or in combination *in vitro* by RNA-seq. **(A–C)** Volcano plots of DEGs in mo-IDECs, comparing the Pam3CSK4 and anti-IgE alone or combination treatment and control groups, respectively. Horizontal lines in the volcano plots were cutoff for -log_10_ (0.05). **(D)** Number of DEGs upregulated or downregulated at each treatment group. **(E)** The Venn diagram represented the overlap of DEGs among different treatment groups. **(F–H)** Heat maps of DEGs relevant to carbon metabolism among different treatment groups, comparing the control group (n = 4).

To investigate how mo-IDECs adapts to Pam3CSK4, anti-IgE, and Pam3CSK4 in combination with anti-IgE, we performed RNA-seq of each treatment group, as well as the unstimulated group. Genes with an | log_2_ FC | > 0 and *p*-value < 0.05 were categorized as differentially expressed. The overall distribution of DEGs is shown in **Figures 4A–E** and **Supplementary Figures S2A–C**. The results revealed that in comparison to the unstimulated group, treatment with Pam3CSK4 and anti-IgE alone or in combination significantly altered 3,890 genes (2,202 upregulated and 1,688 downregulated), 1107 genes (756 upregulated and 351 downregulated), and 3,669 genes (2,038 upregulated and 1,631 downregulated), respectively (**Figure 4D**). The combination treatment group significantly altered 1,607 genes (824 upregulated and 783 downregulated) or 3,078 genes (1,461 upregulated and 1,617 downregulated) compared to the Pam3CSK4 or anti-IgE group, respectively (**Supplementary Figure S2C**). Venn diagram illustrated that 255 genes (145 upregulated and 110 downregulated) were identified in all treated groups (**Figure 4E**, **Supplementary Table S1**). In summary, these data suggested that Pam3CSK4 alone or in combination anti-IgE-treated groups identified more DEGs than the anti-IgE-treated group.

### 3.3 Alteration of carbon metabolism in mo-IDECs after treatment

To recognize in detail the potential metabolic pathways involved in these DEGs, we performed KEGG enrichment analysis. These KEGG pathway analyses were also illustrated as dot diagrams, with the gene ratio denoted by size and the significance denoted by color.

**Figure 5:**
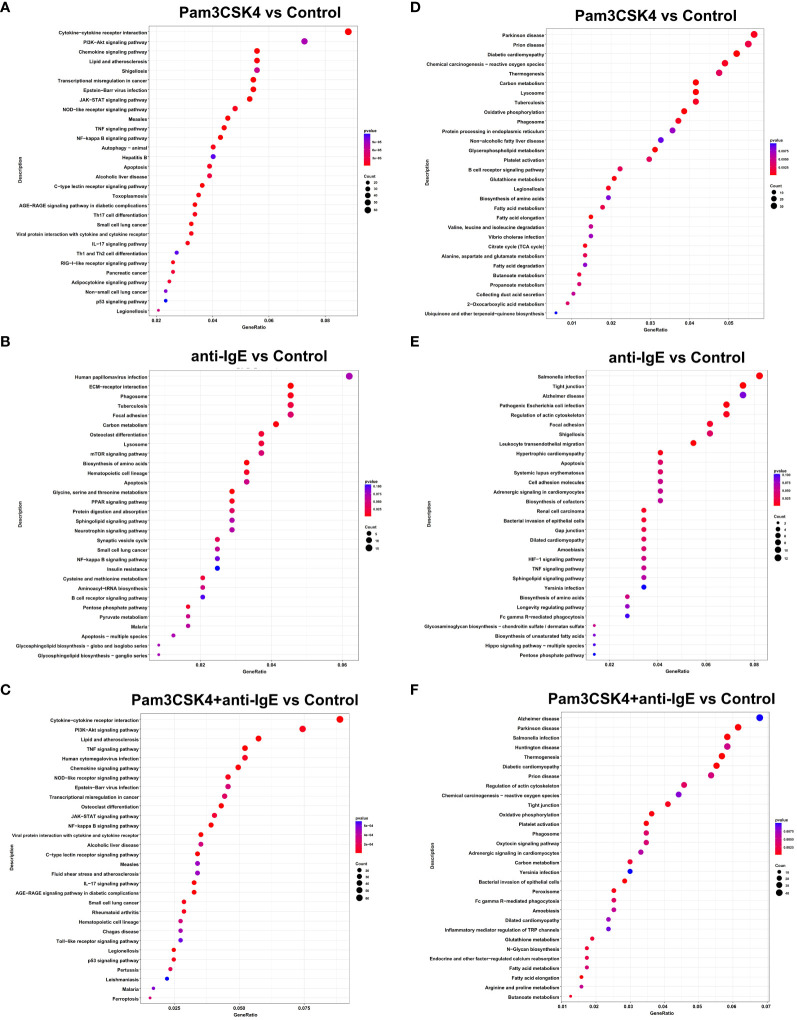
KEGG enrichment analysis of DEGs in mo-IDECs exposed to Pam3CSK4 and anti-IgE alone or in combination *in vitro* based on the data of RNA-seq. **(A–C)** Dots diagram of the top-30 enrichment pathways of upregulated DEGs. **(D–F)** Dots diagram of the top-30 enrichment pathways of downregulated DEGs. DEGs compared the Pam3CSK4 and anti-IgE alone or combination treatment and control groups, respectively. The size of the dots represented the number of the pathway-related genes (count). The *p*-values of each KEGG term are shown by color. The gene ratio described the ratio of the count to the number of all DEGs.

Compared to the unstimulated group, the upregulated DEGs of mo-IDECs treated with Pam3CSK4 and anti-IgE alone or in combination were assigned to 101, 17, and 83 significant KEGG pathways, respectively (*p* < 0.05), and the top 30 pathways for each group are listed in **Figures 5A–C**. We found that mo-IDECs induced by anti-IgE were mainly enriched in glycine, serine, and threonine metabolism, biosynthesis of amino acids, carbon metabolism, pentose phosphate pathway, cysteine and methionine metabolism, and aminoacyl-tRNA biosynthesis compared to unstimulated mo-IDECs, while the metabolic pathways in other groups were not enriched (**Figures 5A–C**). However, downregulated DEGs of mo-IDECs were assigned to 69, 18, and 56 significant KEGG pathways, respectively (*p* < 0.05), and the top 30 pathways for each group are listed in **Figures 5D–F**. The results showed that several metabolic pathways, including carbon metabolism, oxidative phosphorylation, fatty acid elongation, fatty acid metabolism, glutathione metabolism, and butanoate metabolism, were enriched in mo-IDECs treated with Pam3CSK4 alone or in combination. While the biosynthesis of amino acids was the only differentially enriched metabolic pathway in the anti-IgE-treated mo-IDECs, with the biosynthesis of cofactors and unsaturated fatty acids and the pentose phosphate pathway also ranking in the top 30 (**Figures 5D–F**). Collectively, these data indicated that the carbon metabolism of mo-IDECs was differently affected in response to Pam3CSK4 and anti-IgE alone or in combination. Furthermore, we found distinct gene expression profiles related to carbon metabolism (**Figures 4F–H**), suggesting that carbon metabolism varied among the different treatment groups.

In addition, we found that upregulated DEGs of mo-IDECs treated with Pam3CSK4 combined with anti-IgE were assigned to 29 or 94 significant KEGG pathways, compared to Pam3CSK4 or anti-IgE alone, respectively (*p* < 0.05), while downregulated DEGs had 31 or 37 significant KEGG pathways, and the top 30 pathways are listed in **Supplementary Figures S2D–G**. These results further revealed that the carbon metabolism of mo-IDECs was indeed affected in all treatment groups.

### 3.4 Metabolite profiling of mo-IDECs

**Figure 6:**
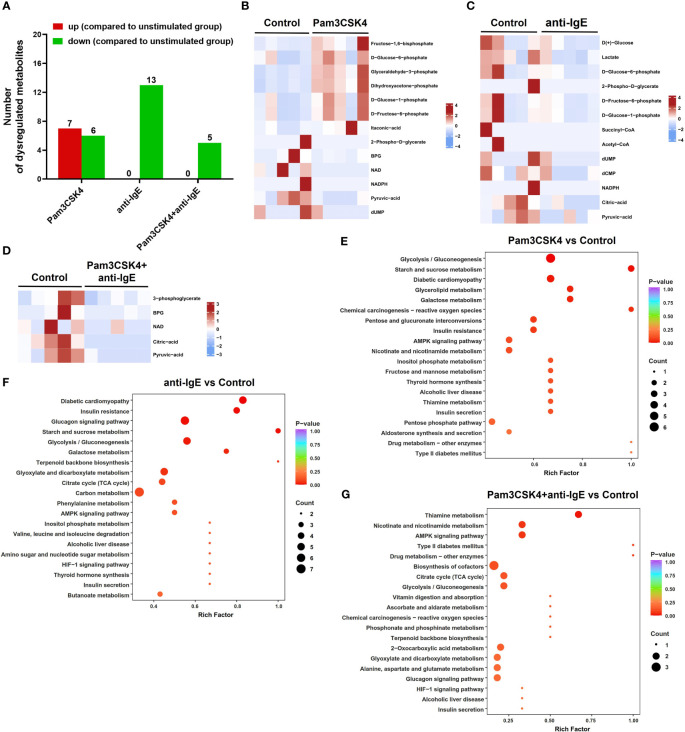
Overview of the metabolite profiling of mo-IDECs in response to Pam3CSK4 and anti-IgE alone or in combination *in vitro* by LC-MS/MS. **(A)** Number of upregulated or downregulated metabolites at each treatment group. **(B–D)** Heat maps represented the differential metabolites among different treatment groups, comparing the control group (n = 5). **(E–G)** KEGG pathway enrichment of differential metabolites in each treatment group compared to control. BPG, 2,3-diphosphoglycerate; NAD, nicotinamide adenine dinucleotide; NADPH, nicotinamide adenine dinucleotide phosphate; dUMP, deoxy-uridine monophosphate; and dCMP, deoxycytidylic acid.

To further investigate metabolomic changes, five samples from each group were selected for targeted energy metabolomic analysis using mo-IDECs stimulated with Pam3CSK4, anti-IgE, and Pam3CSK4 in combination with anti-IgE, as well as unstimulated mo-IDECs. Metabolites with VIP ≥ 1 and fold change ≥ 2 or fold change ≤ 0.5 were identified as differential metabolites. The overall distribution of dysregulated metabolites is shown in **Figure 6**, **Supplementary Figures S3** and **S4**.

The results revealed that in comparison to the unstimulated group, the metabolites of the Pam3CSK4 group (7 upregulated and 6 downregulated), anti-IgE group (0 upregulated and 13 downregulated), and Pam3CSK4 combined with the anti-IgE group (0 upregulated and 5 downregulated) changed significantly (**Figures 6A–D**). The combination treatment group significantly altered 16 metabolites (2 upregulated and 14 downregulated) or 9 metabolites (8 upregulated and 1 downregulated) compared to the Pam3CSK4 or anti-IgE group, respectively (**Supplementary Figures S3A–C**). These data suggested that there were different metabolic mappings in each treatment group.

Moreover, to obtain a comprehensive overview of the differential metabolites, we performed a KEGG pathway enrichment analysis and classified the results according to pathway types. These results showed that the common effect of different treatments on the metabolism of mo-IDECs was mainly manifested in glycolysis/gluconeogenesis in carbon metabolism (**Figures 6E–G**, **Supplementary Figures S3D–E, S4**). These results suggested that glycolysis/gluconeogenesis was altered in mo-IDECs with different treatments.

### 3.5 Activation of glycolysis pathway in treated mo-IDECs

**Figure 7:**
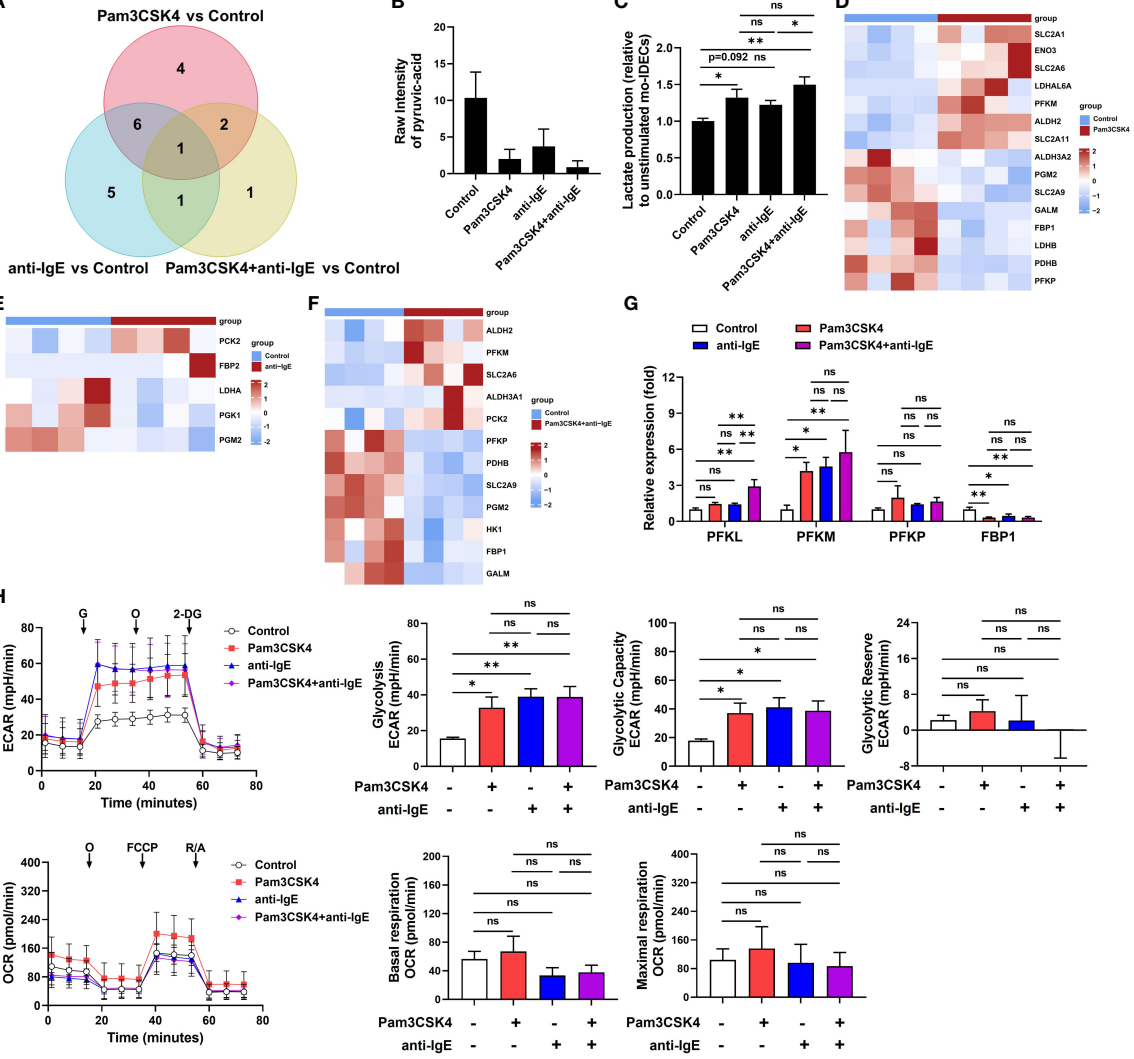
Activation of the glycolysis pathway in mo-IDECs after Pam3CSK4 and anti-IgE alone or in combination, stimulation *in vitro*. **(A)** Venn diagrams represented the overlap of dysregulated metabolites among different treatment groups based on the targeted energy metabolomic analysis. **(B)** Quantification of the pyruvic-acid metabolite in mo-IDECs in control and treatment groups based on the data of targeted energy metabolomic analysis (n = 5). **(C)** Quantification of the lactate content in cell-free supernatants from each treatment group relative to control by fluorometric analysis (n = 4). **(D–F)** Heat maps of DEGs relevant to glycolysis/gluconeogenesis pathway among different treatment groups, comparing the control group by RNA-seq (n = 4). **(G)** The relative mRNA expression of *PFKL*, *PFKM*, *PFKP*, and *FBP1* in mo-IDECs with different treatments were evaluated by qRT-PCR (n = 4). **(H)** Overall rate curves of extracellular acidification rate (ECAR), glycolytic, glycolytic capacity, and glycolytic reserve by seahorse glycolysis stress test (n = 4). **(I)** Overall rate curves of oxygen consumption rate (OCR), mitochondrial basal and maximal respiratory by seahorse cell mitochondrial stress test (n = 4). Data were presented as mean ± SEM. ^ns^ *p* > 0.05, * *p* < 0.05, and ** *p* < 0.01 as determined by one-way ANOVA. G, glucose; O, oligomycin; 2-DG, 2-deoxyglucose; FCCP, carbonyl cyanide-4 (trifluoromethoxy) phenylhydrazone; and R/A, rotenone/antimycin A. ns represents not significant.

To further explore the role of glycolysis/gluconeogenesis in mo-IDECs, through Venn diagram, we found that pyruvic acid is a common differential metabolite (**Figure 7A**). In addition, based on the data of targeted energy metabolomic analysis, results showed that compared to the control group, the levels of pyruvic acid in the Pam3CSK4 and anti-IgE alone or in combination groups significantly decreased to 19.3%, 36%, and 8.5%, respectively, while there was no difference between each treatment group (**Figure 7B**). Pyruvic acid was converted into acetyl-CoA, alanine, and lactate (29), but we did not find an increase in these metabolites within different stimulated mo-IDECs (**Figures 6B–D**, **Supplementary Figures S3B, C**).

To further clarify the reason for the reduction of pyruvic acid, we detected the changes in extracellular lactate and found that there were varying degrees of increase in each treatment group, suggesting a higher conversion of pyruvic acid into lactate (**Figure 7C**). Furthermore, heat maps displayed DEGs related to glycolysis/gluconeogenesis pathway, including genes associated with the rate-limiting enzymes of glycolysis (6-phosphofrucose kinase-1, PFK1) and gluconeogenesis (fructose diphosphatase, FBP) (**Figures 7D–F**). In addition, we analyzed the mRNA expression levels of genes related with PFK1 and FBP enzymes by qRT-PCR, and the results showed that in comparison with the unstimulated group, the expression of *PFKM* was upregulated, while *FBP1* downregulated (**Figure 7G**). In parallel, we performed metabolic flux analysis using seahorse assays. Our analysis showed that compared to the unstimulated group, each treatment group significantly increased glycolysis rate and glycolytic capacity, while there was no difference between each treatment group (**Figure 7H**). At the same time, we found that there were no significant changes in glycolytic reserve and mitochondrial basal and maximal respiratory capacity (**Figures 7H, I**). These data indicated that the glycolysis pathway of mo-IDECs was activated, but mitochondrial function was not completely damaged after treatment with different stimulators.

## 4 Discussion

**Figure 8:**
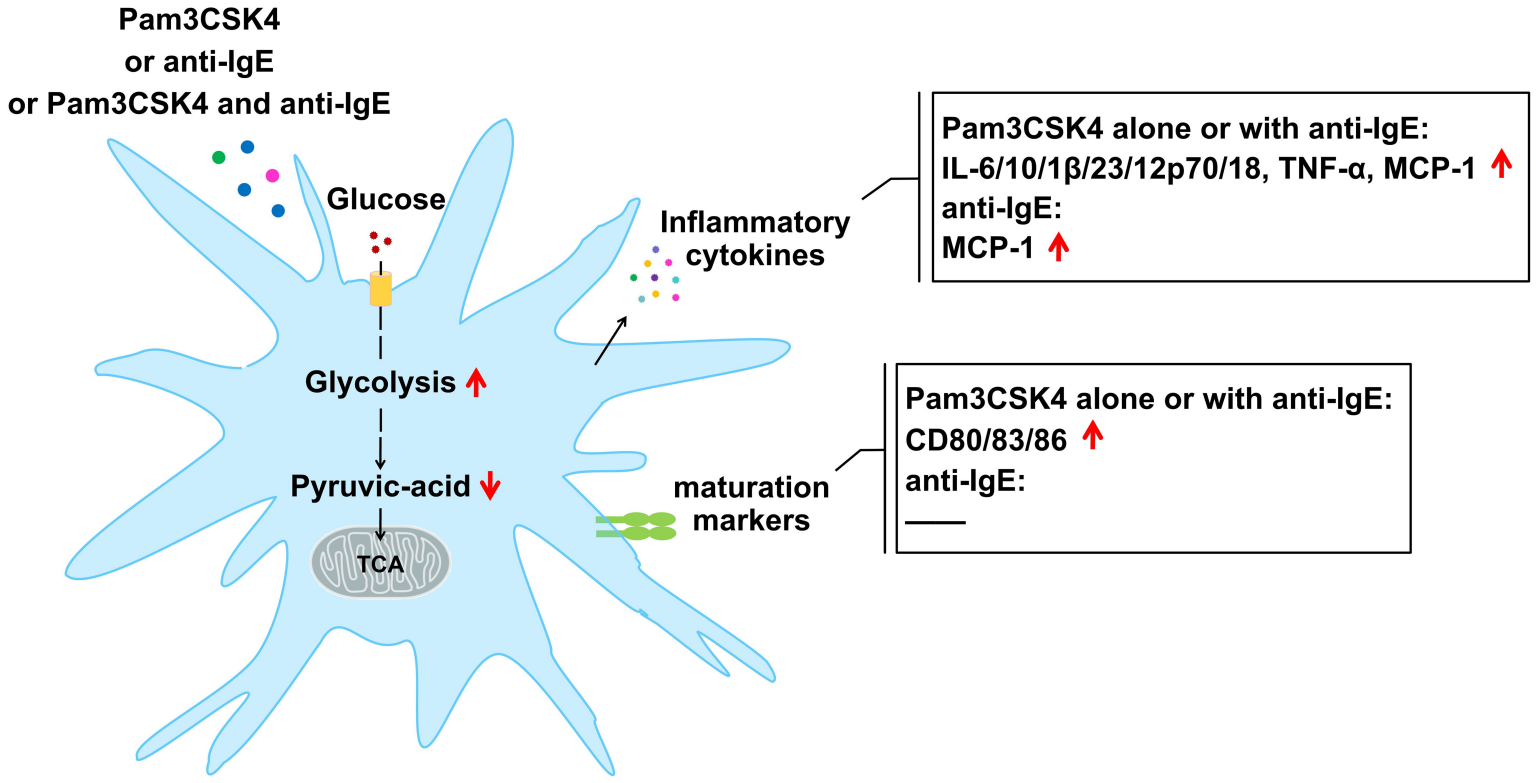
Changes in mature markers, cytokines, and profiles of cellular glucose metabolism in mo-IDECs after treatment with Pam3CSK4 and anti-IgE alone or in combination.

The metabolic reprogramming of cellular energy mediates the function of immune cells (12, 13), yet there is no study yet into the effects of FcϵRI and TLR2 on the metabolism and inflammatory effect function of IDECs from skin lesions of patients with AD. However, due to the low number of IDECs in the skin lesions of patients with AD, we have investigated the expression of maturation markers, the production of cytokines, and cellular energy metabolism in an *in vitro* model of IDECs after treatment with Pam3CSK4 and anti-IgE alone or in combination (**Figure 8**).

Here, by comparison to the control group, we discovered that Pam3CSK4, as a potent stimulator, alone or in combination with anti-IgE significantly increased the expression of maturation markers and the production of inflammatory cytokines, while anti-IgE only promoted the release of inflammatory factors to a lesser extent. Anti-IgE did not affect the activation of mo-IDECs but only weakly promoted the generation of inflammatory factors, which may be due to the internalization or low expression of FcϵRI receptor on the surface of mo-IDECs from healthy donors. It has been reported that the expression of FcϵRI on the surface of DCs could be regulated by a complex “atopic environment” (30). Our results supported previous reports that TLR2 ligation downregulated the expression of FcϵRI α-chain (31, 32) (**Supplementary Figure S1**). In addition, we also found that inflammatory cytokines and maturation markers in Pam3CSK4 combined with anti-IgE group were higher than in the anti-IgE group, while it was not in the Pam3CSK4 group, suggesting that the interaction of FcϵRI and TLR2 in IDECs might not occur, or that TLR2 downregulated the expression of FcϵRI α-chain, which affected the interaction.

In addition, studies have reported that inflammatory cytokines, such as IL-12, IL-18, IL-6, IL-23, and IL-1β, could drive Th1, Th2, and Th17 immune responses (28, 33, 34), and we found that mo-IDECs secreted the above factors, indicating that mo-IDECs could mediate the priming of distinct T-helper cell responses in the presence of Pam3CSK4 or anti-IgE. These results suggested that FcϵRI and TLR2 evoked the inflammatory response of IDECs by upregulating the production of inflammatory cytokines in AD. Furthermore, it has been reported that the skin barrier defect is one of the primary defects in AD. The disorder of the skin barrier is accompanied by skin colonization of microbes, such as *Staphylococcus aureus*, or skin penetration of allergens, thereby promoting the generation of immunoregulatory cytokines. These cytokines induce expression of the inflammatory cytokines, which in turn induce inflammation, barrier disruption, and itch (35–38). Therefore, these findings further confirmed that the skin barrier defect in patients with AD could allow danger signals from bacteria or allergens to amplify inflammation and exacerbate the disease.

Next, we found that carbon metabolism was affected in all treatment groups. Central carbon metabolism plays a leading role in carbon metabolism, which is often called energy metabolism, including glycolysis, oxidative phosphorylation, and pentose phosphate pathway. Furthermore, our results showed that the profiles of energy metabolism in mo-IDECs were different, which might imply that the immunologic function of these cells varied in the presence of microbes and/or allergens. Differential metabolites were co-enriched in the glycolysis/gluconeogenesis pathway, suggesting that the glycolysis/gluconeogenesis pathway in the carbon metabolism of mo-IDECs was affected after treatment. In addition, we found that pyruvic acid was the common differential metabolite and was downregulated to varying degrees compared to the control group. Research reported that pyruvic acid, as the end product of glycolysis, could be reduced into lactate, transaminated into alanine or transported into the mitochondrion to form acetyl-CoA for entry into the tricarboxylic acid (TCA) cycle (29), but we did not find an increase in intracellular lactate/alanine/acetyl-CoA metabolites. At the same time, we found that there were varying degrees of increase in extracellular lactate in each treatment group, suggesting that the reason for the reduction of pyruvic acid might be due to its transformation into lactate, which was secreted into the extracellular space.

It has been reported that cellular glycolysis could be increased by inhibiting the expression of *FBP1* (gluconeogenesis enzyme-related gene) (39) and we found that the expression of *FBP1* was downregulated, suggesting that the glycolysis pathway was activated in all treatment groups. Moreover, we also found that *PFKM* (glycolysis enzyme-related gene) was upregulated and glycolysis rate and glycolysis capacity increased in all treatment groups, while the basic and maximum respiratory capacity of the mitochondria did not, indicating that glycolysis was activated and mitochondrial function was not completely damaged. In addition, we found that intracellular ATP was not a differential metabolite, indicating that cellular energy in mo-IDECs remained unchanged after treatment. At the same time, we also found that itaconic acid, which inhibits glycolysis (40–42), increased in the Pam3CSK4 group. The above results suggested that the glycolysis pathway might be activated to a limited extent for cell survival in mo-IDECs of different treatment groups. Finally, results showed that there were no differences in the level of pyruvic acid, the expression of *PFKM*, *PFKP*, or *FBP1*, and the levels of ECAR or OCR between all treatment groups, suggesting that both FcϵRI and TLR2 in IDECs might mediate cellular glucose metabolism. Researchers have reported that glycolysis was involved in the regulation of inflammation of AD in mice (19–21), and the above findings further supported the role of glycolysis in AD inflammation.

There are several limitations in the current study: (1) all samples were obtained from healthy volunteers without any skin lesions, but it was not ruled out whether these donors had other diseases without symptoms; (2) failure to further confirm cell metabolism using an *in vitro* IDECs model from peripheral blood or *in vivo* IDECs from skin lesions of patients with AD; (3) lack of precise intracellular carbon metabolism flux through glucose C^13^ tracing experiments; (4) lack of experiments studying the effect of blocking glycolysis or blocking FcϵRI or TLR2 receptors on IDECs maturation and inflammation in different treatments; and (5) no observation of dynamic changes in metabolism and inflammatory factors of IDECs from different treatment groups. Future studies are needed to address these limitations.

In conclusion, the glycolysis pathway in IDECs may be activated to upregulate inflammatory factors through FcϵRI or TLR2, thereby driving different T-cell immune responses in AD. The TLR2 agonist Pam3CSK4, as a potent stimulator, has a much higher inflammatory response than anti-IgE. In the presence of both anti-IgE and Pam3CSK4, the interaction of FcϵRI and TLR2 in IDECs may not occur or may be influenced by TLR2 signaling. Future research needs to determine the specific mechanism of inflammatory factor production mediated by the glycolysis pathway and the adaptive immune response of IDECs, which may provide important clues as to how metabolism in IDECs boosts the occurrence and development of AD in the presence of danger signals from bacteria or allergens, and so on.

## Data availability statement

The data presented in the study are deposited in the Genome Sequence Archive for Human repository, accession number HRA007151 and in OMIX, accession number: OMIX006493.

## Ethics statement

The studies involving humans were approved by the Ethics Committee of Southwest Hospital (Approval number KY202258). The studies were conducted in accordance with the local legislation and institutional requirements. The participants provided their written informed consent to participate in this study.

## Author contributions

CG: Writing – original draft, Writing – review & editing. YZ: Writing – original draft, Writing – review & editing. LG: Writing – original draft, Software. WL: Data curation, Writing – original draft. MZ: Writing – original draft, Formal analysis. BN: Writing – review & editing. ZS: Writing – review & editing.
