# Supplementary material for: Distinct maturation, glucose metabolism, and inflammatory function of human monocytes-derived IDECs mediated by anti-IgE and Pam3CSK4 alone or in combination
Source: Front Immunol. 2024 Jul 17;15:1403263. doi: 10.3389/fimmu.2024.1403263 (PMC11288808; doi:10.3389/fimmu.2024.1403263)
Supplement: Supplementary file 1 [file DataSheet_1.pdf]

Supplementary Material

1 Supplementary Data

Figure S1 Pam3CSK4 alone or combination with anti-IgE downregulated the expression of FceRIα in mo-IDECs *in vitro* by flow cytometry. **(A)** Specific gating strategies to identify FceRIα expression on the surface of live mo-IDEC ($CD14^{-}CD1a^{+}$) cells after stimulation with different stimulus for 24 h as measured by flow cytometry (n = 5-6). **(B)** Expression of FceRIα in mo-IDECs. Data were presented as mean ± SEM. Statistical significance were analyzed by the one-way ANOVA. ^{ns} $p > 0.05$, * $p < 0.05$ and ** $p < 0.01$.

Figure S2 Overview of transcriptome profiling of mo-IDECs in response to Pam3CSK4 combination with anti-IgE *in vitro* by RNA-seq. **(A and B)** Volcano plots of DEGs in mo-IDECs, comparing the Pam3CSK4 combination with anti-IgE treatment and Pam3CSK4 and anti-IgE alone groups, respectively. Horizontal lines in volcano plots were cutoff for $-\log_{10} 0.05$. **(C)** Number of DEGs up- or down-regulated at each treatment group. **(D and F)** Dots diagram of the top 30 KEGG enrichment pathways of upregulated DEGs. **(E and G)** Dots diagram of the top 30 KEGG enrichment pathways of downregulated DEGs. The size of the dots represented the number of the pathway related genes (count). The *p*-values of each KEGG term were shown by color. The gene ratio described the ratio of the count to the number of all DEGs.

Figure S3 Overview of metabolite profiling of mo-IDECs in response to Pam3CSK4 combination with anti-IgE *in vitro* by LC-MS/MS. **(A)** Number of up- or down-regulated metabolites at each treatment group. **(B and C)** Heat maps represented the differential metabolites among different treatment groups (n = 5). **(D and E)** KEGG pathway enrichment of dysregulated metabolites. BPG, 2,3-diphosphoglycerate. NADPH, nicotinamide adenine dinucleotide phosphate. UMP, uridine 5'-monophosphate.

Figure S4 KEGG pathway enrichment of dysregulated metabolites in mo-IDECs after different stimulation treatments *in vitro* by LC-MS/MS. **(A-E)** KEGG classification according to the pathway types of dysregulated metabolites.

2 Supplementary Figures

Figure S1

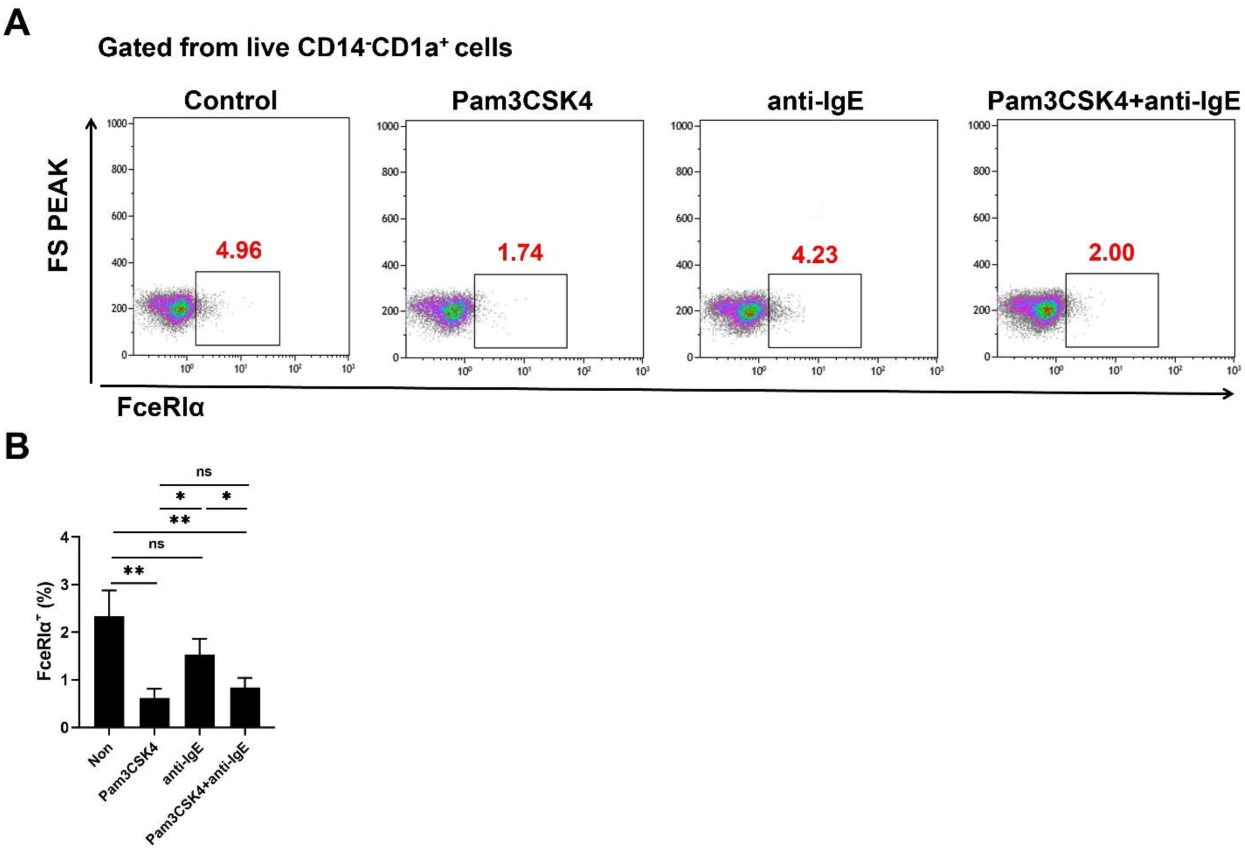
A
Gated from live CD14-CD1a+ cells
Control
Pam3CSK4
anti-IgE
Pam3CSK4+anti-IgE
FS PEAK
4.96
1.74
4.23
2.00
FceRIα
B
FceRIα+ (%)
ns
*
**
Non
Pam3CSK4
anti-IgE
Pam3CSK4+anti-IgE

Figure S2

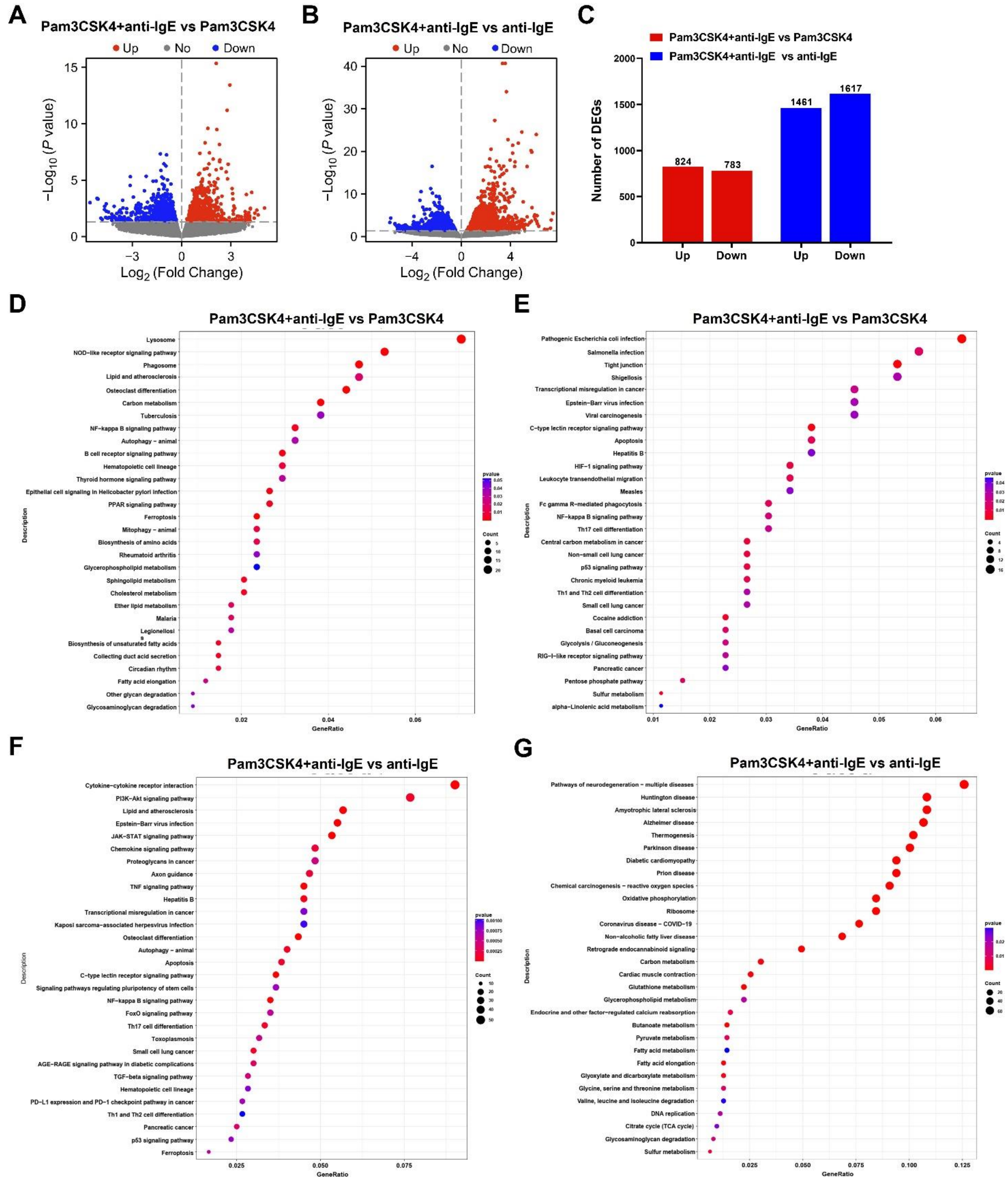

A
Pam3CSK4+anti-IgE vs Pam3CSK4
Up
No
Down
−Log10 (P value)
Log2 (Fold Change)
B
Pam3CSK4+anti-IgE vs anti-IgE
Up
No
Down
−Log10 (P value)
Log2 (Fold Change)
C
Pam3CSK4+anti-IgE vs Pam3CSK4
Pam3CSK4+anti-IgE vs anti-IgE
Number of DEGs
824
783
1461
1617
Up
Down
Up
Down
D
Pam3CSK4+anti-IgE vs Pam3CSK4
Lysosome
NOD−like receptor signaling pathway
Phagosome
Lipid and atherosclerosis
Osteoclast differentiation
Carbon metabolism
Tuberculosis
NF−kappa B signaling pathway
Autophagy − animal
B cell receptor signaling pathway
Hematopoietic cell lineage
Thyroid hormone signaling pathway
Epithelial cell signaling in Helicobacter pylori infection
PPAR signaling pathway
Ferroptosis
Mitophagy − animal
Biosynthesis of amino acids
Rheumatoid arthritis
Glycerophospholipid metabolism
Sphingolipid metabolism
Cholesterol metabolism
Ether lipid metabolism
Malaria
Legionellosis
Biosynthesis of unsaturated fatty acids
Collecting duct acid secretion
Circadian rhythm
Fatty acid elongation
Other glycan degradation
Glycosaminoglycan degradation
Description
GeneRatio
pvalue
Count
E
Pam3CSK4+anti-IgE vs Pam3CSK4
Pathogenic Escherichia coli infection
Salmonella infection
Tight junction
Shigellosis
Transcriptional misregulation in cancer
Epstein−Barr virus infection
Viral carcinogenesis
C−type lectin receptor signaling pathway
Apoptosis
Hepatitis B
HIF−1 signaling pathway
Leukocyte transendothelial migration
Measles
Fc gamma R−mediated phagocytosis
NF−kappa B signaling pathway
Th17 cell differentiation
Central carbon metabolism in cancer
Non−small cell lung cancer
p53 signaling pathway
Chronic myeloid leukemia
Th1 and Th2 cell differentiation
Small cell lung cancer
Cocaine addiction
Basal cell carcinoma
Glycolysis / Gluconeogenesis
RIG−I−like receptor signaling pathway
Pancreatic cancer
Pentose phosphate pathway
Sulfur metabolism
alpha−Linolenic acid metabolism
Description
GeneRatio
pvalue
Count
F
Pam3CSK4+anti-IgE vs anti-IgE
Cytokine−cytokine receptor interaction
PI3K−Akt signaling pathway
Lipid and atherosclerosis
Epstein−Barr virus infection
JAK−STAT signaling pathway
Chemokine signaling pathway
Proteoglycans in cancer
Axon guidance
TNF signaling pathway
Hepatitis B
Transcriptional misregulation in cancer
Kaposi sarcoma−associated herpesvirus infection
Osteoclast differentiation
Autophagy − animal
Apoptosis
C−type lectin receptor signaling pathway
Signaling pathways regulating pluripotency of stem cells
NF−kappa B signaling pathway
FoxO signaling pathway
Th17 cell differentiation
Toxoplasmosis
Small cell lung cancer
AGE−RAGE signaling pathway in diabetic complications
TGF−beta signaling pathway
Hematopoietic cell lineage
PD−L1 expression and PD−1 checkpoint pathway in cancer
Th1 and Th2 cell differentiation
Pancreatic cancer
p53 signaling pathway
Ferroptosis
Description
GeneRatio
pvalue
Count
G
Pam3CSK4+anti-IgE vs anti-IgE
Pathways of neurodegeneration − multiple diseases
Huntington disease
Amyotrophic lateral sclerosis
Alzheimer disease
Thermogenesis
Parkinson disease
Diabetic cardiomyopathy
Prion disease
Chemical carcinogenesis − reactive oxygen species
Oxidative phosphorylation
Ribosome
Coronavirus disease − COVID−19
Non−alcoholic fatty liver disease
Retrograde endocannabinoid signaling
Carbon metabolism
Cardiac muscle contraction
Glutathione metabolism
Glycerophospholipid metabolism
Endocrine and other factor−regulated calcium reabsorption
Butanoate metabolism
Pyruvate metabolism
Fatty acid metabolism
Fatty acid elongation
Glyoxylate and dicarboxylate metabolism
Glycine, serine and threonine metabolism
Valine, leucine and isoleucine degradation
DNA replication
Citrate cycle (TCA cycle)
Glycosaminoglycan degradation
Sulfur metabolism
Description
GeneRatio
pvalue
Count

Figure S3

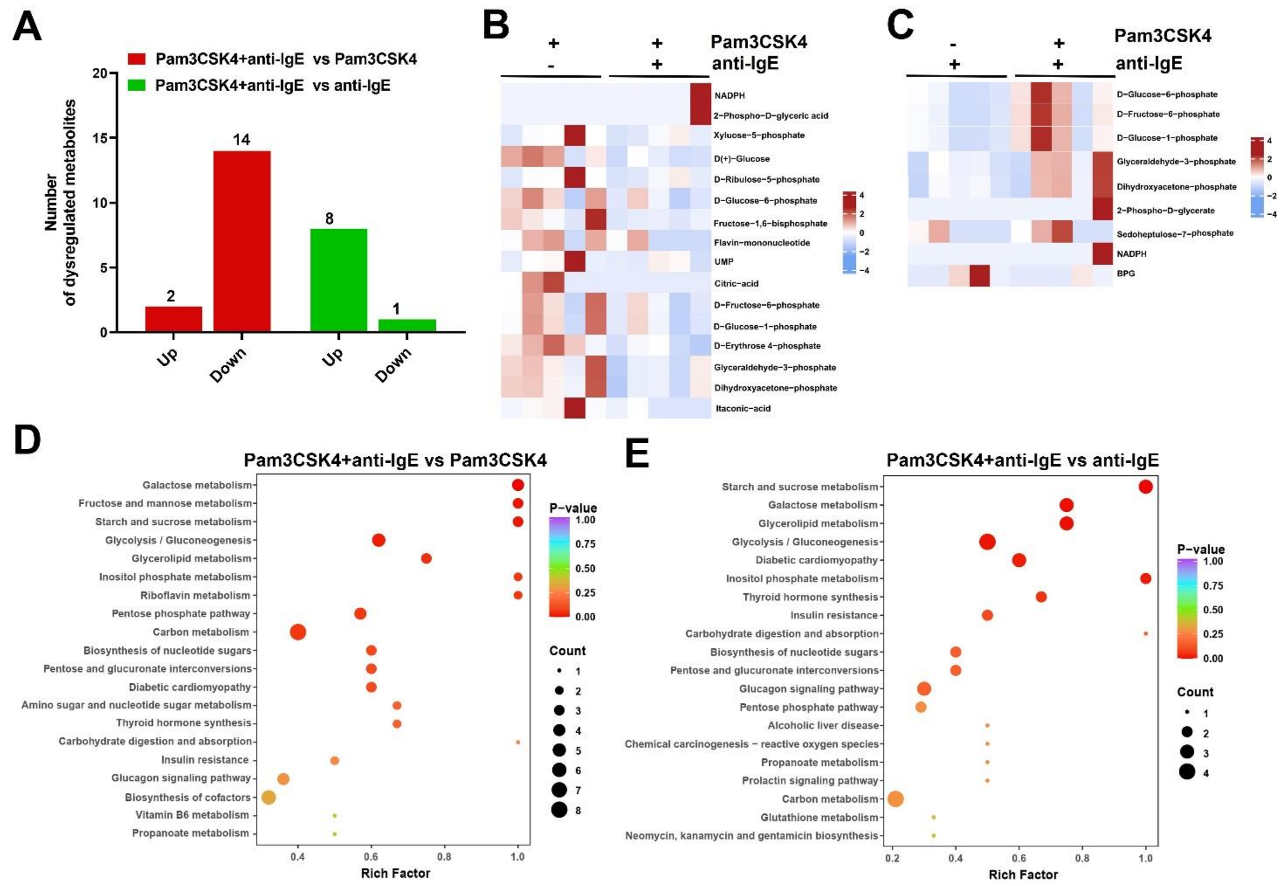
A
Pam3CSK4+anti-IgE vs Pam3CSK4
Pam3CSK4+anti-IgE vs anti-IgE
Number
of dysregulated metabolites
20
15
10
5
0
2
14
8
1
Up
Down
Up
Down
B
+
-
+
+
Pam3CSK4
anti-IgE
NADPH
2−Phospho−D−glyceric acid
Xyluose−5−phosphate
D(+)−Glucose
D−Ribulose−5−phosphate
D−Glucose−6−phosphate
Fructose−1,6−bisphosphate
Flavin−mononucleotide
UMP
Citric−acid
D−Fructose−6−phosphate
D−Glucose−1−phosphate
D−Erythrose 4−phosphate
Glyceraldehyde−3−phosphate
Dihydroxyacetone−phosphate
Itaconic−acid
4
2
0
−2
−4
C
-
+
+
+
Pam3CSK4
anti-IgE
D−Glucose−6−phosphate
D−Fructose−6−phosphate
D−Glucose−1−phosphate
Glyceraldehyde−3−phosphate
Dihydroxyacetone−phosphate
2−Phospho−D−glycerate
Sedoheptulose−7−phosphate
NADPH
BPG
4
2
0
−2
−4
D
Pam3CSK4+anti-IgE vs Pam3CSK4
Galactose metabolism
Fructose and mannose metabolism
Starch and sucrose metabolism
Glycolysis / Gluconeogenesis
Glycerolipid metabolism
Inositol phosphate metabolism
Riboflavin metabolism
Pentose phosphate pathway
Carbon metabolism
Biosynthesis of nucleotide sugars
Pentose and glucuronate interconversions
Diabetic cardiomyopathy
Amino sugar and nucleotide sugar metabolism
Thyroid hormone synthesis
Carbohydrate digestion and absorption
Insulin resistance
Glucagon signaling pathway
Biosynthesis of cofactors
Vitamin B6 metabolism
Propanoate metabolism
0.4
0.6
0.8
1.0
Rich Factor
P−value
1.00
0.75
0.50
0.25
0.00
Count
1
2
3
4
5
6
7
8
E
Pam3CSK4+anti-IgE vs anti-IgE
Starch and sucrose metabolism
Galactose metabolism
Glycerolipid metabolism
Glycolysis / Gluconeogenesis
Diabetic cardiomyopathy
Inositol phosphate metabolism
Thyroid hormone synthesis
Insulin resistance
Carbohydrate digestion and absorption
Biosynthesis of nucleotide sugars
Pentose and glucuronate interconversions
Glucagon signaling pathway
Pentose phosphate pathway
Alcoholic liver disease
Chemical carcinogenesis − reactive oxygen species
Propanoate metabolism
Prolactin signaling pathway
Carbon metabolism
Glutathione metabolism
Neomycin, kanamycin and gentamicin biosynthesis
0.2
0.4
0.6
0.8
1.0
Rich Factor
P−value
1.00
0.75
0.50
0.25
0.00
Count
1
2
3
4

Figure S4

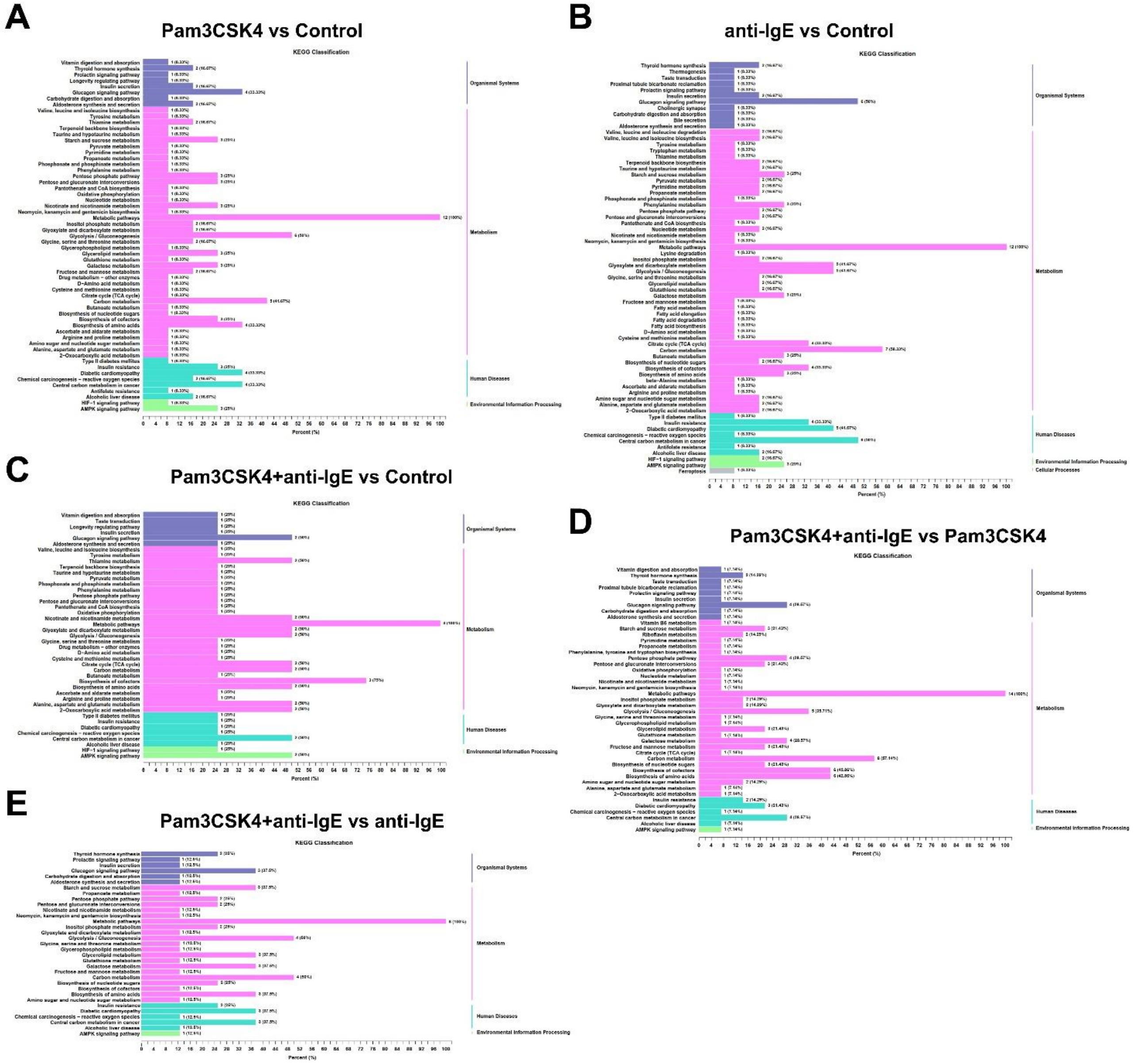
A
Pam3CSK4 vs Control
B
anti-IgE vs Control
C
Pam3CSK4+anti-IgE vs Control
D
Pam3CSK4+anti-IgE vs Pam3CSK4
E
Pam3CSK4+anti-IgE vs anti-IgE
